# Push/Pull Inequality Based High-Speed On-Chip Mixer Enhanced by Wettability

**DOI:** 10.3390/mi11100950

**Published:** 2020-10-21

**Authors:** Toshio Takayama, Naoya Hosokawa, Chia-Hung Dylan Tsai, Makoto Kaneko

**Affiliations:** 1 Department of Mechanical Engineering, Tokyo Institute of Technology, Tokyo 152-8552, Japan; 2 Department of Mechanical Engineering, Osaka University, Osaka 565-0871, Japan; ho_naoya@icloud.com; 3 Department of Mechanical Engineering, National Chiao Tung University, Hsinchu City 30010, Taiwan; dylantsai@nctu.edu.tw; 4 Graduate School of Science and Engineering, Meijo University, Aichi 468-8502, Japan; mkaneko@meijo-u.ac.jp

**Keywords:** push/pull inequality, nonreversible flow pattern, wettability, surfactant, hydrophilic treatment, on-chip mixer

## Abstract

In this paper, a high-speed on-chip mixer using two effects is proposed, i.e., push/pull inequality and wettability. Push/pull inequality and wettability are effective for generating a rotational fluid motion in the chamber and for enhancing the rotational speed by reducing the viscous loss between the liquid and channel wall, respectively. An on-chip mixer is composed of three components, a microfluidic channel for making the main fluid flow, a circular chamber connected to the channel for generating a rotational flow, and an actuator connected at the end of the channel allowing a push/pull motion to be applied to the liquid in the main channel. The flow patterns in the chamber under push/pull motions are nonreversible for each motion and, as a result, produce one-directional torque to the fluid in the circular chamber. This nonreversible motion is called push/pull inequality and eventually creates a swirling flow in the chamber. Using hydrophilic treatments, we executed the experiment with a straight channel and a circular chamber to clarify the mixing characteristics at different flow speeds. According to the results, it is confirmed that the swirling velocity under appropriately tuned wettability is 100 times faster than that without tuning.

## 1. Introduction

Mixing is an important operation for both macro- and micro-scale reactions in various fields, in particular chemistry and biomedical research. The scale of mixing vessels depends on the purpose and utilization of the liquid. For a vessel with a diameter of several centimeters, the mixing of two liquids is relatively easy unless the viscosity of the liquid is high, such as with honey. In general, the degree of difficulty is determined based on the Reynolds number (Re) and is written as Re =UL/μ, where *U*, *L*, and μ are the velocity of the liquid, the representative dimension of the vessel, and the kinematic viscosity coefficient, respectively. For a flow at a low Reynolds number, which often occurs in a microfluidic channel, it is difficult to make a vortex for mixing. This paper focuses on creating a vortex in a chamber attached within a microfluidic channel. The fundamental mixing technique is spontaneous diffusion [1,2,3,4,5]. However, it takes time to reach a state of equilibrium. To speed up the mixing, vortices are often used for more efficient mixing. We can classify conventional studies into two groups: passive and active approaches. The difference between passive and active approaches is whether the vortex is generated naturally without any actuator or actively with an additional actuator. For a passive approach, there are various studies [6,7,8,9,10,11,12,13,14,15,16] in which the common strategy for generating the vortices is to mix two liquids in a microfluidic channel using a special configuration.

**Figure 1:**
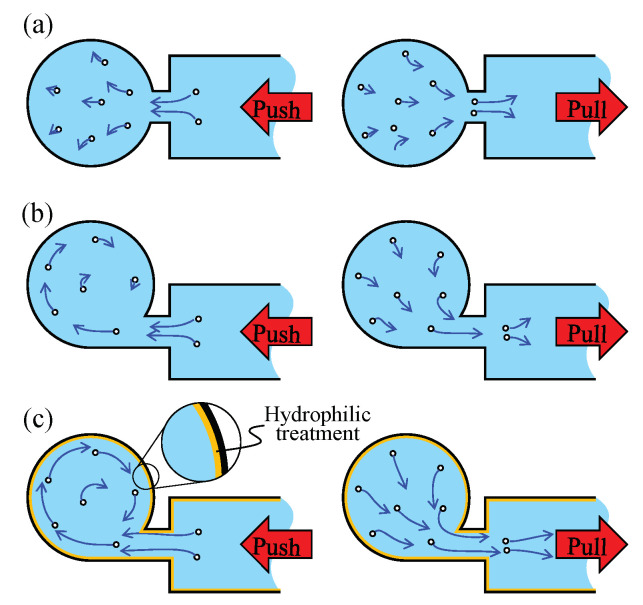
Different scales of swirling motion are introduced under different conditions of push/pull motion: flow patterns (**a**) for a symmetric design, (**b**) an asymmetric design, and (**c**) an asymmetric design with hydrophilic treatment.

We can further classify an active approach into two groups, an indirect active approach in which an active motion is imparted indirectly from the outside of the chip and a direct active approach, where an active motion is given directly to the fluid within the channel. For an indirect active approach, Oberti et al. and Ahmed et al. succeeded in enhancing the mixing efficiency of a laminar flow by applying a vibrational force caused by a loudspeaker [17,18,19]. In addition, Hayakawa et al. proposed a control method for the flow around the pillars by shaking the chip using piezo actuators and succeeded in controlling the motion of the particles among the pillars using the generated vortices [20]. Shang et al. generated a vortex by pushing the surface of the chip using two piezo actuators stuck in a circular chamber [21]. For a direct active approach, Suzuki et al. and Sasaki et al. developed built-in actuators to efficiently mix a liquid [22,23]. Hansen et al. developed microvalves and micropumps driven by the liquid pressure and succeeded in rotating the liquid in a ring-shaped mixer [24]. Glasgow et al. and Niu et al. enhanced the mixing of a laminar flow by applying a periodic perturbation directly to the injected liquid [25,26]. We proposed a vibration-based virtual vortex gear (VVVG or V${}^{3}$G), where a swirling motion can be generated in a microchamber with a diameter of 300 μm through periodic push/pull motions by a piezo actuator directly connected to the microchannel [27], as shown in Figure 1a,b, having geometrical symmetry and asymmetry with respect to the connected part of the neck channel, respectively. Owing to the geometrical symmetry, the flow pattern is close to reversible, as shown in Figure 1a, whereas it is a nonreversible flow pattern in the case of Figure 1b. When the flow pattern is reversible, we cannot expect a swirling flow with the scale of the chamber diameter because the net torque around the center of the circular chamber is nearly zero in the time average for a single push/pull motion. By contrast, when the flow pattern is nonreversible, we can expect a large swirling flow in the circular chamber because the net torque for the liquid is no longer zero for one cycle of the push/pull motion. A “push/pull inequality based mixer” is based on this characteristic. As one feature of a push/pull inequality-based mixer, we can create a swirling flow in the circular chamber under a periodic push/pull action without generating any net flow in the main channel. As a result, there is no concern regarding the loss of liquid in the main channel. In addition, the wettability is effective at increasing the rotational speed in the chamber by reducing the viscous loss between the liquid and channel wall, as shown in Figure 1c [28]. The swirling velocity under the appropriate wettability with hydrophilic treatment is 100 times faster than that without hydrophilic treatment.

The remainder of this paper is organized as follows. In Section 2, we describe the basic working principle for generating the swirling motion using the push/pull inequality and wettability in detail. In Section 3, we detail the experimental setup and procedures. In Section 4, we show the experimental results. In Section 5, we discuss the relationship between the contact angle and the swirling velocity. We also discuss the maximum frequency through a comparison between the linear channel and the circular chamber. Finally, we provide some concluding remarks and areas of future research in Section 6.

## 2. Basic Working Principle of the High-Speed On-Chip Mixer

The push/pull inequality and wettability are effective for generating the rotational fluid motion in a chamber and for enhancing this speed by reducing the viscous loss between the liquid and channel wall, respectively.

### 2.1. Push/Pull Inequality Based Swirling Flow

Figure 1 shows the basic concept of a push/pull inequality based swirling flow in a circular chamber, where we suppose that an actuator is implemented at the end of the main fluidic channel. Figure 1a,b shows two examples of the chamber connection to the main fluidic channel, where they are geometrically symmetrical and asymmetric with respect to the connected part of the neck channel, respectively. Push/pull actions are imparted by the actuator. Owing to the geometrical symmetry, the flow pattern is close to reversible in the case of Figure 1a, whereas in the case of Figure 1b, it is a nonreversible flow pattern. When the flow pattern is reversible, we cannot expect a swirling flow with the scale of the chamber diameter because the net torque around the center of the circular chamber is nearly zero in the average time for a single push/pull motion. By contrast, when the flow pattern is nonreversible, we can expect a large swirling flow in the circular chamber because the net torque for the liquid is no longer zero for one cycle of push/pull motion. It should be noted that an inertia effect coming from a liquid mass may also enhance the nonreversible flow pattern under a high push/pull frequency. Implementing a closed air chamber also contributes to increasing the swirling flow [27]. Such a nonreversible flow pattern can be further enhanced by the design of the microfluidic chip, in particular by the angle of the connecting channel with respect to the tangential line of the circular chamber. Two-dimensional models are shown in Figure 1, and the tendency of a nonreversible flow pattern is more complicated under a 3D model than under a 2D model. This is the working principle of push/pull inequality based swirling motion.

### 2.2. Wettability Enhanced Swirling Flow Velocity

Figure 1c shows a simple illustration under a wet surface condition where Figure 1b,c shows the same mechanical configuration except for the surface wettability. Suppose that the surface in Figure 1c is wetter than that in Figure 1b. Under such a condition, the swirling flow velocity is faster in Figure 1c than that in Figure 1b.

**Figure 2:**
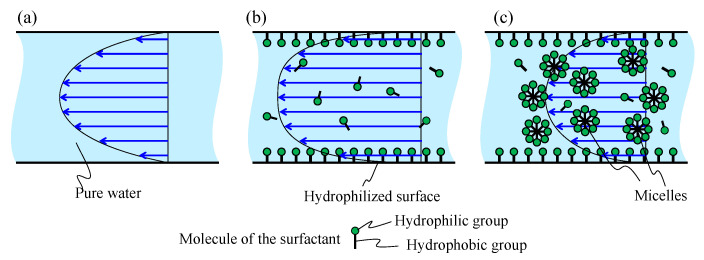
Flow patterns under different liquid conditions: (**a**) pure water, (**b**) water under a surfactant, and (**c**) water under a surfactant with numerous micelles.

Figure 2 shows the principle of reducing the viscous force between the liquid and the polydimethylsiloxane(PDMS) inner surface covered by the surfactant, in the case of a continuous flow in a straight micro channel. Each arrow shows the local flow velocity, and we can realize the flow pattern based on all arrows. When the molecules of the surfactants cover the inner surface of the PDMS, the viscous force is reduced, and the mean flow velocity consequently increases under the same driven pressure. For example, the mean velocity under the surfactant increases, as illustrated in Figure 2b, compared with that under pure water, as shown in Figure 2a.

For a higher concentration of the surfactant, the liquid results in an overcritical micelle state where the surfactant increases the micelle viscosity, as shown in Figure 2c [29]. As a result, the mean flow velocity decreases because of the increase in flow resistance. According to this phenomenon, mixing with the surfactant in the proposed push/pull system will increase the efficiency of the mixing owing to the enhanced flow rate, as illustrated in Figure 2b. However, the mixing efficiency may be oppositely reduced if micelles are formed owing to an overly concentrated surfactant in the liquid. This tendency suggests that there is an optimum concentration for generating the maximum velocity for the liquid motion as far as such a surfactant is utilized. Coating by a hydrophilic agent may work better than the surfactant because it does not produce any micelles. Based on these discussions, in this study, we use both a surfactant and a hydrophilic agent to enhance the mixing speed.

## 3. Experimental Setup and Procedure

### 3.1. Experimental Setup

**Figure 3:**
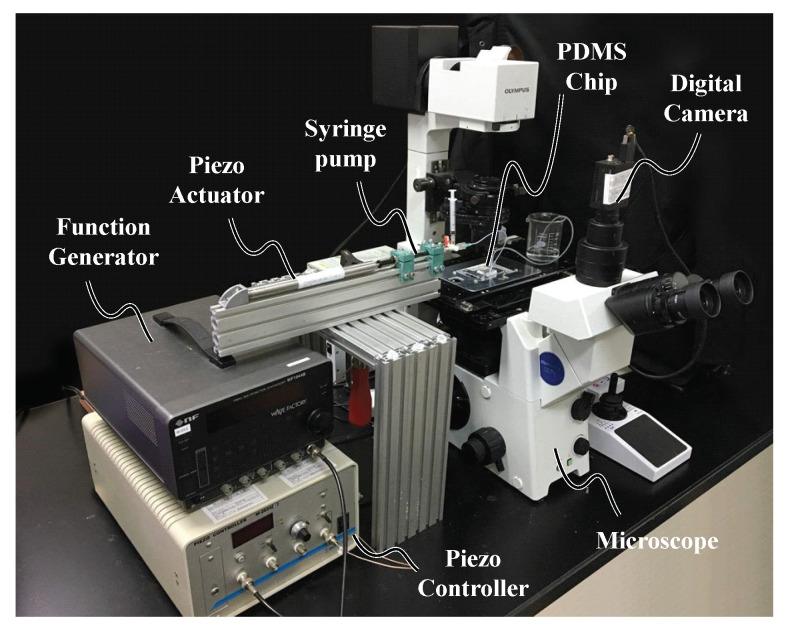
Experimental setup.

Figure 3 shows the experimental setup, where a square wave of 0.3 Vp-p is generated by the function generator (NF Corporation, WF1944B, Yokohama, Japan), which is amplified 15 times using a piezo controller (MESS-TEK, M-2655S) and sent to the piezo actuator (MESS-TEK, PSt 150/5/40 VS10, Wako, Japan) to push and pull the liquid periodically using a syringe pump. The syringe pump and PDMS chip were connected using a PTFE tube to transmit the push and pull forces to the liquid in a microfluidic channel. The microscope was equipped with a digital high-speed camera (Photron, IDP-Express R2000, Tokyo, Japan) and used to observe microbeads mixed in the liquid for a flow visualization. The mixed microbeads (Invitrogen DVB/Carboxyl 1 μm) were made of polystyrene. To maintain the same experimental conditions, the same number of droplets created by the dropper bottle of the microbeads was mixed with the same volume of the solutions in the microtubes. Furthermore, the density data were evaluated through a unification of the initial value such that we can suppress the effect of the error while mixing both the liquid and microbeads. The high density of the microbeads changes the fluid characteristics, and the low density makes it difficult to observe the change in luminance. As a result, optimal conditions exist for the number of microbeads, which we determined through trial and error.

**Figure 4:**
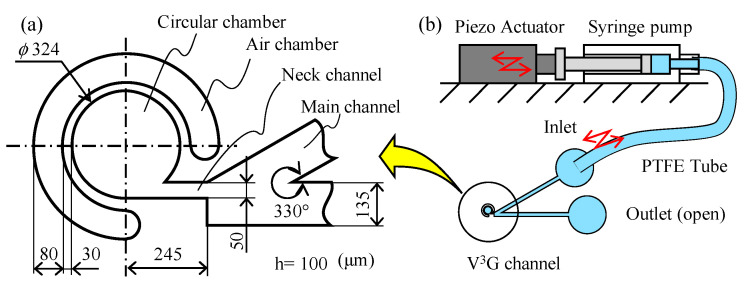
Design of the microfluidic channel and schematic diagrams. (**a**) Channel dimensions. (**b**) The flow is driven by a piezo actuator through a syringe pump.

Figure 4a,b shows the channel design and the schematic diagram including the connection of the piezo actuator, syringe pump, PTFE tube, and microfluidic channel. The microfluidic channel was made using a conventional photolithographic technique. A mold made using SU-8 was cast with polydimethylsiloxane (PDMS) and bonded to a glass slide. The height of the channel was 100 μm. Here, V${}^{3}$G consisted of a V-shaped main channel, a narrow neck channel, a circular chamber, and an air chamber.

We used polysorbate 80 (NOF Corporation) as the surfactant and Lipidure-CM5206 (NOF Corporation, Tokyo, Japan) as the hydrophilic coating agent. These materials are usually used for actual experiments in biomedical fields [30,31,32]. Because polysorbate 80 can be used without damaging the cells at up to 0.2 wt/v%, we used 0.1 wt/v% and 0.2 wt/v% liquids to observe the difference in the effect of the micelles [33]. To coat the PDMS surface using Lipidure-CM5206, we dissolved 0.5 wt/v% of Lipidure in ethanol and mixed the solution and toluene at a 1:1 ratio. The Lipidure mixture was injected into the channel and volatilized for coating onto the surface of the channel.

### 3.2. Experimental Procedure and Evaluation Method

First, the entire area, except for the air chamber, was filled with the solution. Next, a solution containing a high concentration of microbeads of diameter 1 μm was injected into the main channel. The piezo actuator was then actuated to generate a swirling flow. The high-concentration solution in the main channel was gradually mixed up to the circular chamber, and the concentration in the circular chamber increased.

To confirm the repeatability and remove the influence of small errors in the chip manufacturing, we executed the experiments three times under the same conditions, including the materials and frequency applied, i.e., pure water, a 0.1 wt/v% surfactant solution, a 0.2 wt/v% surfactant solution, and hydrophilic-coated channels and frequencies of 400, 600, 800, and 1000 Hz, respectively.

**Figure 5:**
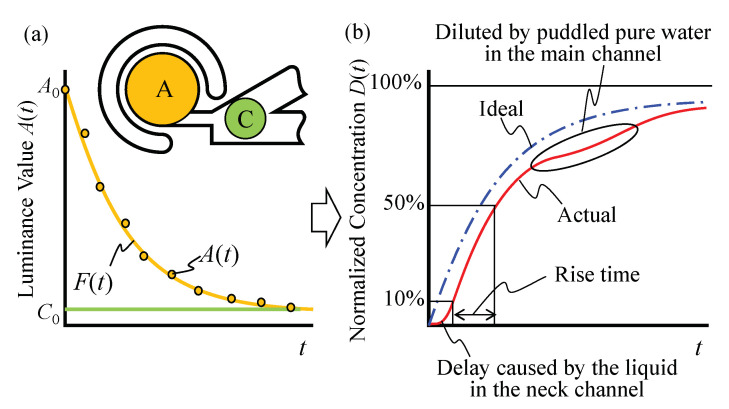
Evaluation method of the mixing speed: (**a**) measured luminance value and (**b**) normalized concentration.

Figure 5 shows the evaluation procedure of the mixing speed from the luminance values of Regions A and C. By increasing the amount of microbeads inside the chamber, The luminance value of Region A gradually decreases as shown in the experiments shown later. If the volume of the main channel is sufficiently large compared to that of the circular chamber, the luminance of Region A will become the same as that of Region C. We can thus obtain a normalized concentration through the following equation:

(1) $$D\left(t\right)=\frac{1-A\left(t\right)/A_{0}}{1-C_{0}/A_{0}}$$

where D(t), A(t), $A_{0}$, and $C_{0}$ are the normalized concentration, the measured discrete luminance value of Region A, the initial luminance value of Region A, and the initial luminance value of Region C, respectively. Ideally, *D*(*t*) increases monotonically like an ideal line, as the dash-dotted line in Figure 5b indicates. During this experiment, we manually injected microbeads into the main channel, and thus, the flow speed was not exactly the same for each experiment. This caused a difference in the number of microbeads that invade the neck channel under the initial condition. This caused a difference of D(t) in the rising delay. Moreover, outside the view area of the microscope, puddles of pure water occasionally remained, and while applying the vibrational force, such puddles moved and diluted the concentration of the entire area, as the actual line in Figure 5b shows. Thus, we assumed that this is a tertiary delay system to evaluate the mixing speed numerically, and we fit A(t) to the following function F(t) using the least-squares method:

(2) $$F\left(t\right)=a_{0}\exp\left(-a_{1}t\right)+a_{2}\exp\left(-a_{3}t\right)+a_{4}\exp\left(-a_{5}t\right)$$

where $a_{i}\left(i=0∼5\right)$ are the coefficients obtained. Thus, we evaluate the mixing speed by using the rise time, which is defined by D(t) from 10% to 50% and is calculated by F(t) instead of A(t). This is the same approach used in evaluating the rise time for an electric circuit.

## 4. Experimental Results

**Figure 6:**
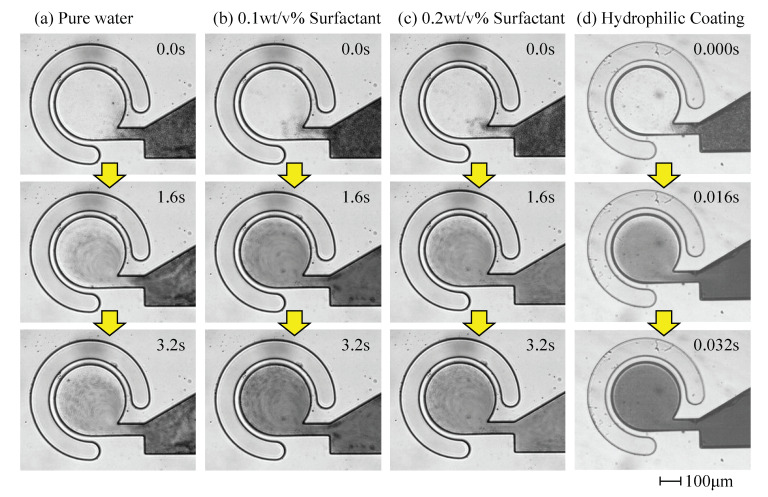
Captured images of the mixing experiment. (**a**) Results of pure water. (**b**) Results of 0.1 wt/v% of surfactant. (**c**) Results of 0.2 wt/v% of surfactant. (**d**) Results of hydrophilic coated channel.

**Figure 7:**
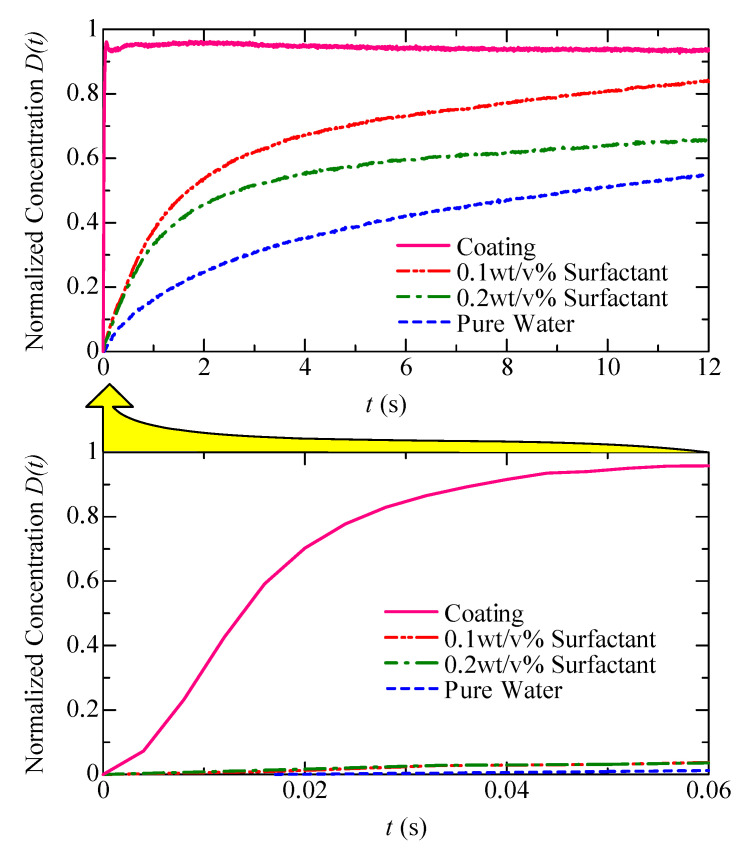
Normalized concentration D(t) (Equation (1)) with respect to time, where the horizontal and vertical axes represent time and D(t), respectively.

Figure 6 shows the images captured during the experiment under a pumping frequency of 600 Hz, where Figure 6a–d indicate the mixing results of pure water, 0.1 wt/v% surfactant solution, 0.2 wt/v% surfactant solution, and the hydrophilic coated channel, respectively. It should be noted that the time scale for the hydrophilic coated channel is 100 times smaller than that of the other three materials. This simply means that the swirling velocity for the hydrophilic coated channel is 100 times faster than that of the other three materials. Figure 7 shows the normalized concentration D(t) (Equation (1)) with respect to time, where the horizontal and vertical axes indicate the time and D(t), respectively. We can also see clearly that the swirling velocity for a hydrophilic coated channel is much faster than that for other materials.

**Figure 8:**
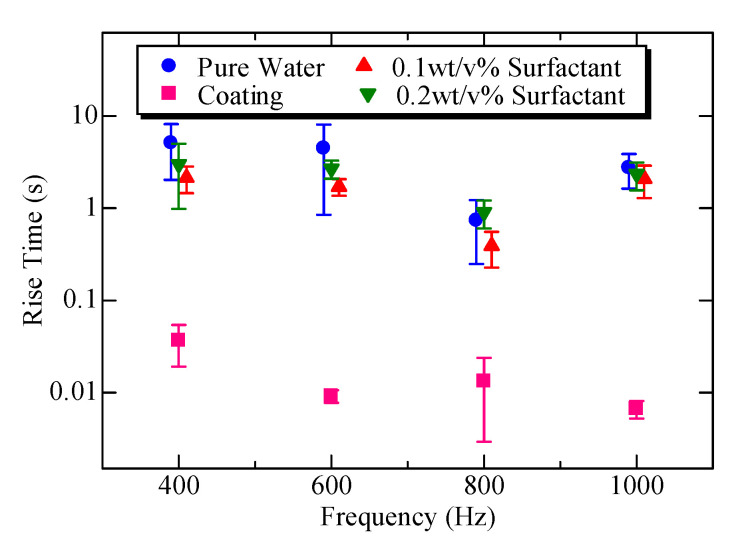
The relationship between the rise time and applied frequencies for four different hydrophilic treatments.

Figure 8 shows the relationship between the rising time and applied frequencies for the four different hydrophilic treatments. From Figure 8, we can also confirm that the quickest response is under the coated channel, and the second, third, and fourth quickest responses are under 0.1 wt/v% surfactant, 0.2 wt/v% of surfactant, and pure water, respectively. Once again, the mixing speed under the coated channel is the fastest among the four experimental conditions and is roughly 100 times faster than that of pure water.

## 5. Discussions

Let us discuss three topics, i.e., the contact angle, which is the index of wettability, the effect of the micelles on the liquid flow, and a comparison between the circular chamber and straight-line fluidic channel.

**Figure 9:**
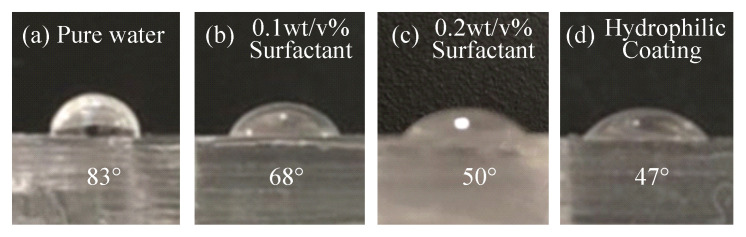
Contact angles for four materials: (**a**) pure water on polydimethylsiloxane(PDMS), (**b**) 0.1 wt/v% surfactant solution on PDMS, (**c**) 0.2 wt/v% surfactant solution on PDMS, and (**d**) pure water on PDMS coated by the hydrophilic material.

In conjunction with the wettability, we measured the contact angle for all materials on a PDMS plate. The microchannel is surrounded by PDMS except for the bottom surface, which is made of glass. If the width of the channel is sufficiently large compared to the height of the channel, the effect of the bottom surface reaches close to 1/2. The width and height of the main channel used are 135 and 100 μm, respectively. The contact area of the PDMS wall was larger than that of the glass wall. Therefore, we measured the contact angle of the solutions only up to the PDMS. Figure 9 shows the contact angles of each solution when placing 20 μL of the solutions on a PDMS plate, where Figure 9a–d show pure water on a PDMS plate, a 0.1 wt/v% surfactant solution on a PDMS plate, a 0.2 wt/v% surfactant solution on a PDMS plate, and pure water on the hydrophilic coated PDMS plate, respectively. When the concentration of the surfactant becomes higher, the contact angle becomes clearly lower, whereas there is no clear difference between the results of the 0.2 wt/v% surfactant solution and that of the hydrophilic coated PDMS.

**Figure 10:**
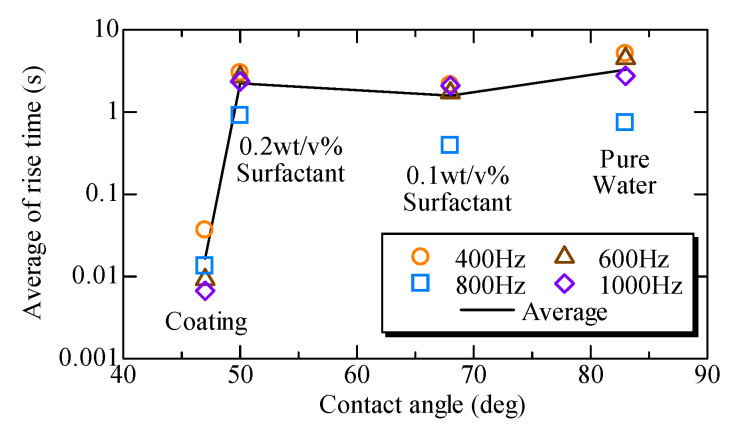
Relationship between the rise time and the contact angle.

It is interesting to know how the correlation between the rise time and the contact angle occurs. Figure 10 shows the relationship between the rise time and the contact angle, where the horizontal and vertical axes represent the contact angle and the rise time, respectively. The rise time remains almost constant for pure water, the 0.1 wt/v% surfactant solution, and the 0.2 wt/v% surfactant solution, whereas the rise time dramatically reduces under the hydrophilic coating. Figure 10 shows that the rise time is less correlated with the contact angle, but is significantly influenced by whether an appropriate coating exists on the inner surface of the microfluidic chip. According to the data sheet, the critical micelle concentration was 0.016 w/v%, which means that both surfactants with 0.1 w/v% and 0.2 w/v% are already over the critical micelle concentration [34]. Furthermore, there should be more micelles under a surfactant with 0.2 w/v% than those under a surfactant with 0.1 w/v%, which generates more drag force in a microchannel under a surfactant with 0.2 w/v% than under a surfactant with 0.1 w/v%. Under such conditions, the rise time under the surfactant with 0.2 w/v% can be less than that under a surfactant with 0.1 w/v%. Therefore, during our experiment, micelles that increase the viscosity may be created. Note that the appearance of the micelles does not reflect the contact angle, which is a static parameter. We believe that this is why the rise time jumps 100 times between the coating and surfactant with 0.2 w/v%.

There are two ways to enhance the swirling velocity in the circular chamber under the given microfluidic channel: choosing the coating material or choosing an appropriate surfactant with the optimum percentage. For the coating material, we can avoid the appearance of micelles, which increases the drag force in the microchannel, although this takes an additional procedure for coating the inner surface. For the surfactant, the key point is to find the optimum concentration percentage that is less than the critical micelle concentration.

The important key words of this study are “flow in microfluidic channel”, “vibration input”, “surfactant”, and “experiment”. However, there are many studies discussing the flow in a microfluidic channel through the use of a “simulation” [7,11,31,35,36,37,38,39,40,41]. The closest study from the viewpoint of overlapping key words is that conducted by Kelder et al., who discussed the mathematical formulation of a fluid flow by considering the “vibration input” and the “surfactant” and showed various interesting “numerical simulations,” while focusing on the behavior of droplets appearing from the membrane rather than those of the fluid flow [31]. Lei et al. discussed the “numerical simulation” of a boundary-driven acoustic streaming based “vibration input” in microfluidic channels and demonstrated a standing wave generated by a harmonic vibration of the boundary vibration input, although the main target was not focused on a fluid flow [41]. Based on a survey of conventional studies, no simulations indicated by the three key phrases “flow in a microfluidic channel”, “vibration input”, and “surfactant” have been conducted. In this study, we observed the wall vibration according to the vibration input, which is due to the wall elasticity. This physical phenomenon suggests that we consider the wall elasticity as well, which makes it even more difficult to see a good fit between the simulation and experiment. This may be a good research topic for a future study.

For the third topic, in which we compare the relationship between the circular chamber and the straight-line fluidic channel, see Appendix A.

## 6. Concluding Remarks

Under the assumption that the periodic push/pull action is given at the input side of the main channel, We showed that the swirling flow pattern in a chamber connected to the main channel can be changed depending on the geometrical parameter. By using the push/pull inequality characteristics, we succeeded in generating a swirling flow with a diameter comparable to that of the microchamber. We confirmed that the push/pull inequality is effective for producing such a large-scale swirling flow. Furthermore, we confirmed that materials such as surfactant solutions and hydrophilic coatings enhance the swirling velocity because these materials contribute to decreasing the contact friction between the liquid and the surface of the microchannel. Through experiments, we confirmed that the combination of a push/pull action and hydrophilic coating resulted in a mixing speed 100 times faster than that without a coating. We also found that the optimum frequency for maximizing the swirling speed is 800 Hz. We should note that the idea proposed in this study can be applied not only to a particular microchannel, but also for general microchannels combined with periodic push/pull motions. In the future, we plan to apply the proposed technique to multiple chambers for a quick concentration control to achieve an on-chip micro medicine dispensing machine. Moreover, suppose there are two time constants for the reaction, one of which is the cell time constant $T_{c}$ for the changing characteristics and the other is the time constant $T_{l}$ for mixing the two liquids. To evaluate the cell characteristics appropriately, we should maintain $T_{c}>>T_{l}$. To maintain this condition, $T_{l}$ should be kept as small as possible to maintain a safety margin. The results of this work will contribute to opening a new research area in which we can observe the reaction of the cells against medicines whose time constant is small. Therefore, confirming that the proposed method does not affect the growth of the actual cells will also be an area of future study.

## Figures and Tables

**Figure A1:**
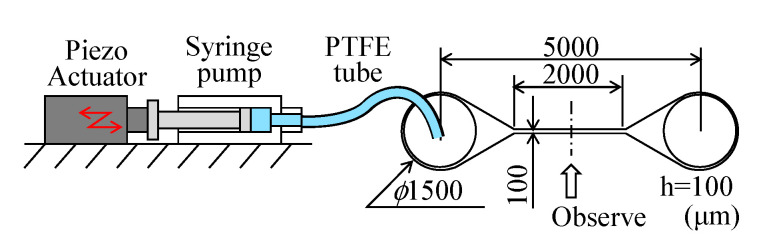
The channel design of the straight-line channel.

**Figure A2:**
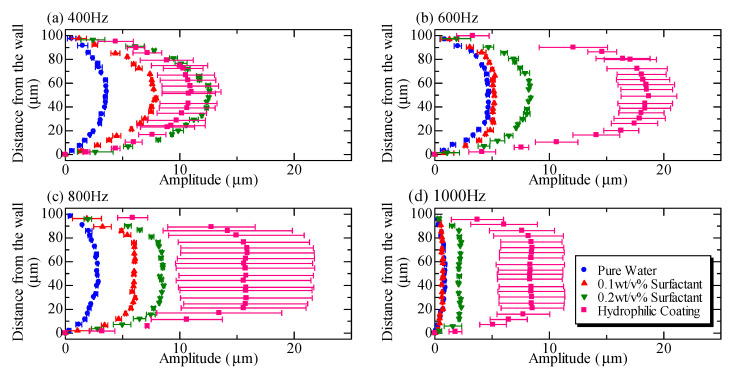
Amplitude profile in the straight-line channel when a vibrational force is applied at frequencies of (**a**) 400, (**b**) 600, (**c**) 800, and (**d**) 1000 Hz.

**Figure A3:**
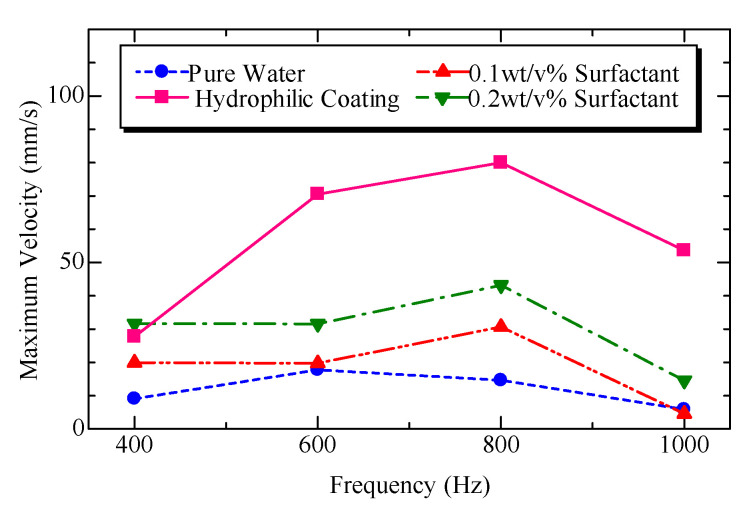
Maximum velocities in a straight-line channel when a vibrational force is applied under various frequencies.

## Appendix A. Comparison between the Circular Chamber and the Straight-Line Fluidic Channel

Another interesting question is how the amplitude profile looks like when applying various materials. To confirm the effect of these hydrophiles on the flow when applying a vibrational force, we measured the amplitude of the liquid in a straight-line channel of 100 μm in width by using a star-trail method [28]. The channel design is shown in Figure A1.

Figure A2 shows the measured amplitude by utilizing microbeads. As an interesting observation, we can also confirm a large amplitude under the surface coating using a hydrophilic coating for all frequencies except at a frequency of 400 Hz. As another interesting result, the amplitude increases with respect to the increase in frequency and decreases after the peak under a frequency of 600 Hz. In the sense of maximizing the amplitude, the optimum frequency is 600 Hz. We should note that the variation in the maximum velocity is the highest under a frequency of 800 Hz. Because the velocity is computed by the product of the amplitude and frequency, we can easily convert from the amplitude to the maximum velocity by utilizing the equation v=2πAf, where *v*, *A*, and *f* are the maximum velocity of the microbeads, the amplitude of the sinusoidal motion of the beads, and the frequency, respectively. Figure A3 shows the maximum velocity converted from the amplitude, where the horizontal and vertical axes represent the frequency and the maximum velocity, respectively. Figure A3 indicates that the maximum velocities become the fastest under a frequency of 800 Hz and under the hydrophilic coating. This means that the velocity is the most effectively enhanced under these conditions. Moreover, it shows that the surfactant also contributes to enhancing the velocities. We wonder if 800 Hz is perhaps one of the resonance frequencies, and we can see noises with a significant fluctuation. There are many unpredictable factors, such as the elasticity distribution of the PDMS and the unknown dynamics of the tubes connected to the chip. However, we cannot identify what causes such dynamic characteristics.

The maximum speed occurs at 600 and 1000 Hz for a circular chamber and at 800 Hz for a straight-line channel. Coincidentally, the frequency of 800 Hz leads to the maximum fluid velocity in both a straight-line channel and a circular chamber.

For a straight-line channel, for the maximum velocity, we can confirm that the first, second, third, and fourth fastest motions are under a coated channel, 0.2 wt/v% surfactant, 0.1 wt/v% surfactant, and pure water, respectively at a frequency between 600 and 1000 Hz, whereas the order of 0.1 wt/v% surfactant and 0.2 wt/v% surfactant was the opposite for the circular chamber. This is perhaps caused by the difference in the increase in the viscosity of the micelles. Another possible reason is the different mechanical configuration, where the width of the narrowest part of the microfluidic channel is 50 and 100 μm, respectively. Although a reversal occurs between a circular chamber and a straight-line channel, the difference is insignificant.
